# Cell wall skeleton of *Mycobacterium bovis* BCG enhances the vaccine potential of antigen 85B against tuberculosis by inducing Th1 and Th17 responses

**DOI:** 10.1371/journal.pone.0213536

**Published:** 2019-03-08

**Authors:** Yong Woo Back, Seunga Choi, Han-Gyu Choi, Ki-Won Shin, Yeo-Jin Son, Tae-Hyun Paik, Hwa-Jung Kim

**Affiliations:** Department of Microbiology, and Department of Medical Science, College of Medicine, Chungnam National University, Daejeon, Republic of Korea; Cornell University, UNITED STATES

## Abstract

A safe and effective adjuvant is necessary to induce reliable protective efficacy of the protein-based vaccines against tuberculosis (TB). Mycobacterial components, such as synthetic cord factor and arabinogalactan, have been used as one of the adjuvant components. *Mycobacterium bovis* bacillus Calmette- Guérin cell-wall skeleton (BCG-CWS) has been used as an effective immune-stimulator. However, it is not proven whether BCG-CWS can be an effective adjuvant for the subunit protein vaccine of TB. In this study, we demonstrated that the BCG-CWS effectively coupled with Ag85B and enhanced the conjugated Ag85B activity on the maturation of dendritic cells (DCs). Ag85B-BCG-CWS-matured DCs induced significant Th1 and Th17 responses when compared to BCG-CWS or Ag85B alone. In addition, significant Ag85B-specific Th1 and Th17 responses were induced in Ag85B-BCG-CWS-immunized mice before infection with *M*. *tuberculosis* and maintained after infection. Moreover, Ag85B-BCG-CWS showed significant protective effect comparable to live BCG at 6 weeks after infection and maintained its protective efficacy at 32 weeks post-challenge, whereas live BCG did not. These results suggest that the BCG-CWS may be an effective adjuvant candidate for a protein-based vaccine against TB.

## Introduction

Tuberculosis (TB) is a major infectious disease that causes 1.3 million deaths per year worldwide [1]. The reactivation risk of the latent TB in an immunosuppressed individual and the advent of multidrug-resistant TB have made the control of TB difficult [2–4]. Vaccination is one of the best methods to control infectious diseases. The only TB vaccine, *Mycobacterium bovis* bacillus Calmette-Guérin (BCG), is unable to significantly prevent adult pulmonary TB and the reactivation of dormant TB [5]. Research on the protein-based vaccines have been focusing on a reliable candidate for BCG-prime boosting. Because a protein alone induces a weak immune response, the adjuvants or viral vectors have been used as an antigen delivery system. Therefore, an adjuvant with a strong immunostimulatory effect is essential to develop these subunit vaccines. In recent years, the multistage TB subunit vaccines using new adjuvant systems, such as IC31, AS01, and GLA-SE, are currently under clinical development [6]. However, better adjuvants that are safe and effective in humans must be developed.

Most adjuvants are Toll-like receptor (TLR) agonists and are in a liposomal or emulsion form mixed with the antigens. Among the TB vaccines, AS01 used in the M72 vaccine is a monophosphoryl lipid A (MPL, TLR-4 agonist)-based adjuvant and liposomal formulation combined with a saponin [6]. IC31 is an adjuvant containing the TLR-9 agonist and is used in vaccines H56 and H4. It is also reported that ID93 combined with a synthetic TLR-4 agonist (glucopyranosyl lipid adjuvant) formulated in a stable oil-in-water emulsion (GLA-SE) elicits significant protection against TB [7]. Mycobacterial components such as synthetic cord factor (TDB) [8], arabinogalactan (AG) [9], and arabinomannan [10] have been included as one of the adjuvant components. We have previously demonstrated that the BCG-cell wall skeleton (BCG-CWS) is a potent immune-adjuvant that promotes antibody production and Th1-skewed immune responses against a conjugated antigen [11]. The clinical efficacy of the BCG-CWS as a tumor immunotherapeutic agent has been demonstrated [12, 13]. It is known that BCG-CWS induces DC maturation via the TLR-2 and TLR-4 pathways [14].

Appropriate DC activation is essential to induce the Th1 immune responses which play a major role in anti-mycobacterial immunity. We hypothesized that the BCG-CWS could be an adjuvant or delivery vehicle for the subunit protein vaccines against TB. It is also possible that the direct conjugation of the proteins with BCG-CWS specifically activates the antigen presenting cells that uptake the target protein, compared to the mixing of the protein with an adjuvant [11]. In the present study, we investigated the DC maturation activity and the vaccine efficacy of BCG-CWS conjugated with Ag85B, which is one of the antigens frequently used in clinical trials of TB vaccines. Ag85B-BCG-CWS induced the Th1 and Th17 responses via DC maturation and provided significant long-term TB protection in a mouse challenge model.

## Materials and methods

### Animals

Six to eight-week-old female C57BL/6 mice were purchased from Nara biotech (Seoul, Korea). All animals were maintained under specific pathogen-free (SPF) barrier conditions at the Preclinical Research Center (PCRC) of Chungnam National University Hospital, Daejeon, Korea. Mice were maintained as per the guide for care and use of the Korean Food and Drug Administration (KFDA). All animal studies conducted were approved by the Ethics Committee and Institutional Animal Care and Use Committee (Permit number 2014-0197-3) of the Laboratory Animal research Center at IACUC (CNU-00284) of Chungnam National University (Daejeon Korea).

### Bacteria

*Mycobacterium tuberculosis* (Mtb) H37Ra (ATCC 25177) was purchased from American Type Culture Collection (ATCC, Manassas, VA). *Mycobacterium* bovis BCG (Tokyo strain) was kindly provided by the Korean Institute of Tuberculosis (KIT). All mycobacteria were grown in 7H9 medium supplemented with 0.5% glycerol, 10% oleic acid, albumin, dextrose, and catalase (OADC; BD Biosciences, San Jose, CA, USA).

### Purification of recombinant Ag85B protein from *Escherichia coli*

To produce recombinant Ag85B protein, the corresponding gene was amplified by PCR using Mtb H37Rv ATCC 27294 genomic DNA as a template and the primer sequences 5′-CATATGACAGACGTGAGCCGAAAG-3′ (forward) and 5′- AAGCTTGCCGGCGCCTAACGAACTCTG-3′ (reverse). The Ag85B gene was inserted into a pET23b (+) plasmid (Novagen, Madison, WI, USA), and the resultant products were sequenced. The recombinant protein was expressed in *E*. *coli* BL21 cells and purified as described previously [15, 16]. Finally, the purified endotoxin-removed recombinant protein was filter-sterilized and frozen at -70 °C. The protein concentration was estimated using a bicinchoninic acid protein assay kit (Pierce, Rockford, IL, USA) with bovine serum albumin as a standard.

### Coupling of Ag85B to BCG-CWS

BCG-CWS was prepared as described previously [11]. The prepared BCG-CWS was suspended in 100% 2- propanol (50 mg/mL, dry weight) and stored at −20 °C until use. The Ag85B protein was covalently coupled to carboxyl groups in BCG-CWS by a two-step N-hydroxysulfosuccinimide (NHS)-enhanced 1-ethyl-3-(3-dimethylaminopropyl)carbodiimide hydrochloride (EDC)-mediated coupling procedure as previously described with slight modification [11]. Briefly, 500 μg of BCG-CWS resuspended in coupling buffer (20 mM EDC and 50 mM NHS dissolved in 100% 2-propanol) was conjugated with Ag85B at different concentrations (pH 7.4) by mixing end-to-end overnight at 4 °C. The unbound protein was removed by washing three times with suspension buffer (0.02% Tween 80 and 1% ethanol in PBS). The resulting Ag85B-conjugated BCG-CWS pellet was resuspended in suspension buffer for protein assays and immunizations experiments.

The amount of protein antigen conjugated to the BCG-CWS was determined by a BCA assay and calculated after subtracting the protein content of unconjugated BCG-CWS. The coupling efficiency was calculated by dividing the total amount of antigen bound to the BCG-CWS by the amount of antigen used in the coupling reaction. The conjugation of the protein with the BCG-CWS was confirmed by SDS PAGE gel and western blot analysis using mouse anti-Ag85B Ab.

### Generation of mouse bone marrow-derived dendritic cells

Bone marrow-derived dendritic cells (BMDCs) were differentiated *in vitro* from isolated bone marrow cells of uninfected 5-6-week old C57BL/6 mice. The cells were generated and cultured as described previously [17]. Briefly, using the BMDC differentiation method, bone marrow cells collected from mouse femurs and tibias were incubated for 7 days in RPMI media supplemented with 10% fetal bovine serum (Lonza), 100 unit/mL penicillin/streptomycin (Lonza), 0.1 mM nonessential amino acids (Lonza), 50 μM β-mercaptoethanol (Lonza), 1 mM sodium pyruvate (Sigma), 20 ng/mL GM-CSF (CreaGene, Gyeonggi, Republic of Korea), and 10 ng/mL IL-4 (CreaGene, Gyeonggi, Republic of Korea).

### Cytokine assays

A sandwich enzyme-linked immunosorbent assay (ELISA) was used to detect IL-6, IL-1β, TNF-α, IL12p70, IL-10, IL-17A, IL-4, IL-23 (eBioscience, San Diego, CA, USA) and IFN-γ (BD Biosciences) levels in culture supernatants as previously described [18].

### Flow cytometry analysis of cell surface molecule expression

BMDCs (2.5 × 10^5^ cells) were isolated by positive selection using CD11c and CD11b microbeads and stimulated for 24 h with Ag85B, MPL (Sigma), MPL+Ag85B, BCG-CWS, or Ag85B-BCG-CWS or medium only. Cells were then stained for cell surface markers using the following antibodies: PE-conjugated anti-CD80, anti-CD86, anti-H-2Kb (MHC class I), and anti-I-Ab (MHC class II) with FITC-conjugated anti-CD11c and Percp Cyanine 5.5-conjugated anti-CD11b antibodies from eBioscience for 30 min at 4 °C. The fluorescence was measured by flow cytometry (FACS).

### *In vitro* T-cell response assay

BMDCs prepared from C57BL/6 mice were stimulated with Ag85B (2.5 μg), MPL (5 μg), MPL+Ag85B (2.5 μg Ag85B with 5 μg MPL), BCG-CWS (5 μg), or Ag85B-BCG-CWS (2.5 μg Ag85B conjugated with 5 μg BCG-CWS) for 24 h. After washing with fresh medium, the cells were seeded into 24-well cell culture plates. CD4^+^ T cells were isolated using a MACS column (Miltenyi Biotec, Bergisch Gladbach, Germany) from splenocytes of naive mice. Stimulated BMDCs were added to the wells that contained CD4^+^ T cells (5 × 10^6^ cells) at a ratio of 1:10 (BMDCs: CD4^+^ T cells) and were cultured for 1 day or 5 days. The supernatants or cells were harvested, and cytokine production was analyzed by ELISA or FACS.

### Acute toxicity test

Six week-old mice (n = 3 animals/group) were given a subcutaneous injection with Ag85B (2.5 μg), BCG-CWS (5 μg), or Ag85B-BCG-CWS (2.5 μg Ag85B conjugated with 5 μg BCG-CWS), and the control was injected with PBS. Each mouse was monitored for changes in body weight for 14 days.

### Flow cytometric analysis and intracellular cytokine staining

The single-cell suspensions from lung cells, the spleen cells, and the cervical lymph were prepared as previously described [15] and then were stimulated with Ag85B (10 μg/mL) for 12 h at 37 °C in the presence of GolgiStop (BD Biosciences). The cells were stained with fluorochrome-conjugated antibodies against anti-CD4-percp (eBioscience), fixed and permeabilized using a Cytofix/Cytoperm kit (BD Biosciences), and stained with anti-IL-17-alexa488 (eBioscience), anti-IFN-γ-PE (BD bioscience) and anti-IL-10-BV510 (BD Biosciences) as previously described [15]. Intracellular cytokine levels were detected on the FACSCanto II with FACSDiva and analyzed using the FlowJo software (Tree star, Ashland, OR, USA).

### *In vivo* experiments, vaccination, Mtb infection in mice, and cell preparation

The 6-week-old female C57BL/6 mice were immunized subcutaneously 3 times at 2 week intervals with 0.2 mL of Ag85B (2.5 μg), BCG-CWS (5 μg), or Ag85B-BCG-CWS (2.5 μg Ag85B conjugated with 5 μg BCG-CWS). In the next experiments, at the time of the first subunit vaccination, one group of mice received a single dose of BCG (1 × 10^4^ CFU) or heat-killed BCG (1 × 10^4^ CFU) injected subcutaneously at the base of the tail.

The mice were challenged with Mtb H37Ra 4 weeks after the last immunization. Briefly, following anesthetization using a xylazine:zoletil (9:1) mixture, 6 mice per group were intratracheally infected with 50 μL suspensions to achieve initial infectious doses of 1 × 10^6^ CFU of H37Ra per mouse lung. Each 3 mice per group were euthanized by CO_2_ chamber method 4 weeks after the final vaccination or 6 weeks after infection to analyze the immune response. Single-cell suspensions from the lung, spleen, and cervical lymph node of each group of mice were stimulated with Ag85B (10 μg/mL) for 24 h or 12 h. Cells were harvested from each group, and the T cell subtype populations were assessed using flow cytometry. The supernatants were harvested, and cytokine production was analyzed by ELISA. These each 3 mice per group were sacrificed by CO_2_ chamber 6 or 32 weeks after infection. Number of bacteria in the spleen or lung was determined by serial 3-fold dilutions of individual whole-organ homogenates in duplicate on 7H10 medium. Organs from the BCG-vaccinated animals were grown in a medium supplemented with 2 μg of 2-thiphenecarboxylic acid hydrazide/mL to selectively inhibit the growth of the residual BCG bacteria in the test organs. Colonies were counted after 2–3 weeks of incubation at 37 °C. Protective efficacies are expressed as log_10_ bacterial counts in immunized mice compared to the bacterial count in the infection controls.

### Histopathology

To analyze the histologic changes, the lung tissues were fixed in 10% formalin, and embedded in paraffin blocks. The lung sections were prepared and stained with hematoxylin and eosin (H&E) and acid-fast bacilli (AFB) staining. All tissue analyses were performed through blind assessment. To quantify the granuloma area and bacterial count, histological images were analyzed using the ImageJ software (National Institutes of Health, Bethesda, MD), as described previously [19].

### Statistical analyses

All results were analyzed using GraphPad Prism 5 software (GraphPad Software, San Diego, CA, USA). The significance of the differences between three or more groups were evaluated using one-way ANOVA followed by Tukey’s multiple comparison analysis. The data in the graphs are expressed as the mean values ± SD; *p < 0.05, **p < 0.01, and ***p < 0.001 were considered statistically significant.

## Results

### Conjugation of Ag85B to BCG-CWS

**Fig 1:**
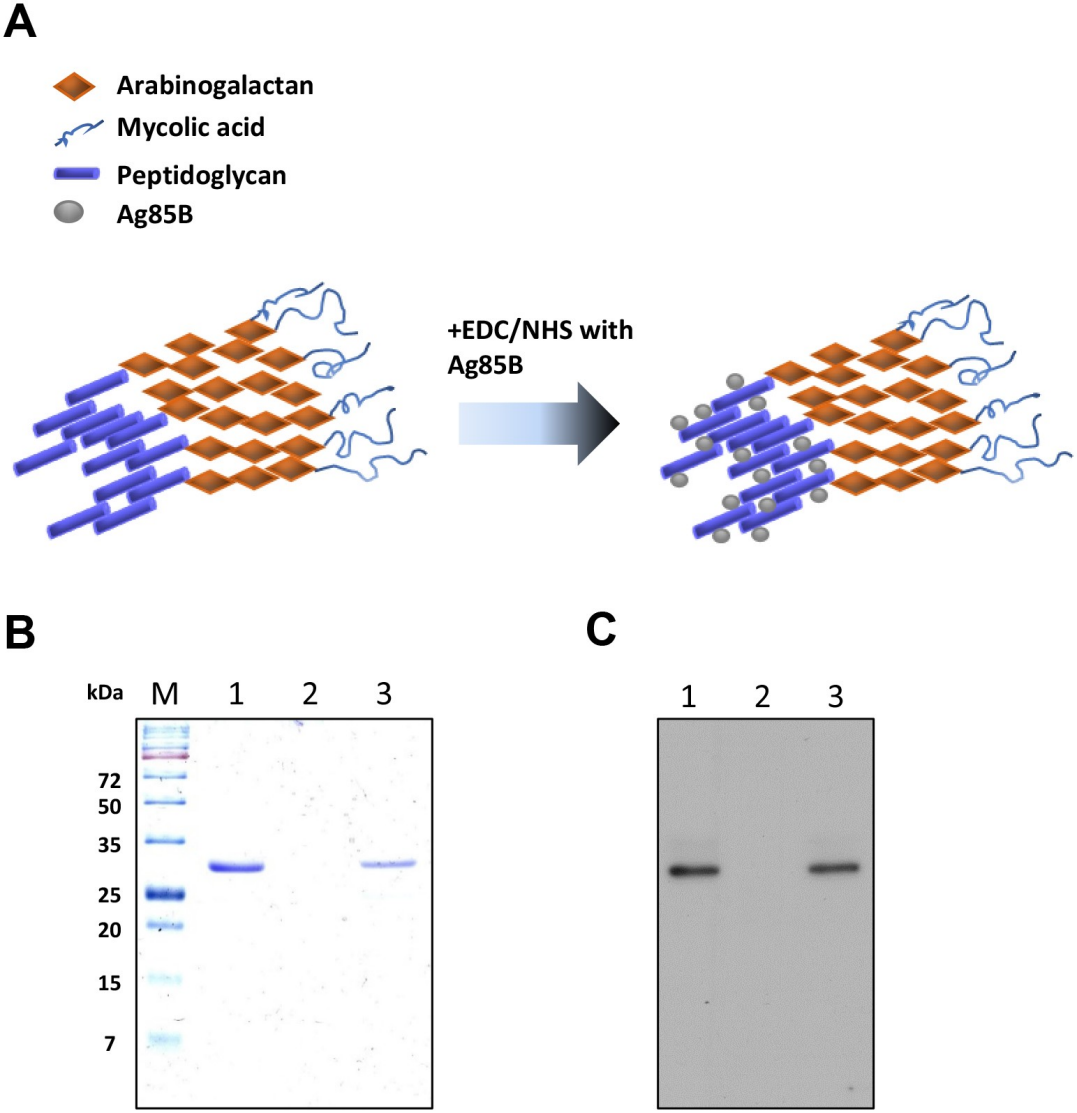
SDS-PAGE and western blot analysis of the conjugation of Ag85B to BCG-CWS. (A) The predicted structure of the conjugation of Ag85B to BCG-CWS. (B) Lane M, molecular weight standard; lane 1, Ag85B (5 μg); lane 2, BCG-CWS (10 μg); lane 3, Ag85B-BCG-CWS (5 μg/10 μg). Protein bands were visualized by Coomassie blue staining. (C) Western blot analysis using mouse anti-Ag85B Ab.

The BCG-CWS, a covalently linked mycolic acid, arabinogalactan, and peptidoglycan complex, has free carboxyl groups from its tetrapeptide amino acids, which can be coupled with the free amino group of a protein [20]. Previously, we demonstrated that bovine serum albumin or ovalbumin are efficiently covalently coupled to a carboxyl group of BCG-CWS, which strongly enhanced the humoral responses against the conjugated proteins [11]. In this study, Ag85B was coupled with the CWS using EDC/NHS to improve the conjugating efficiency (Fig 1A). The protein concentration of the BCG-CWS itself was 0.020 μg/mL. When 500 μg of BCG-CWS was conjugated with Ag85B (500 μg) and then resuspended in 1 mL of suspension buffer, the protein concentration conjugated to the CWS was 0.281 μg/mL, suggesting that BCG-CWS and Ag85B coupled at a ratio of approximately 2:1. The SDS-PAGE analysis confirmed that the Ag85B protein band was detected in the Ag85B-BCG-CWS as a band identical in size to that of the recombinant Ag85B (Fig 1B), but such a band was not observed in the uncoupled BCG-CWS. Conjugation of Ag85B to BCG-CWS was further confirmed by an immunoblot assay using the anti-Ag85B antibody (Fig 1C).

### Ag85B-BCG-CWS induces maturation of DCs

**Fig 2:**
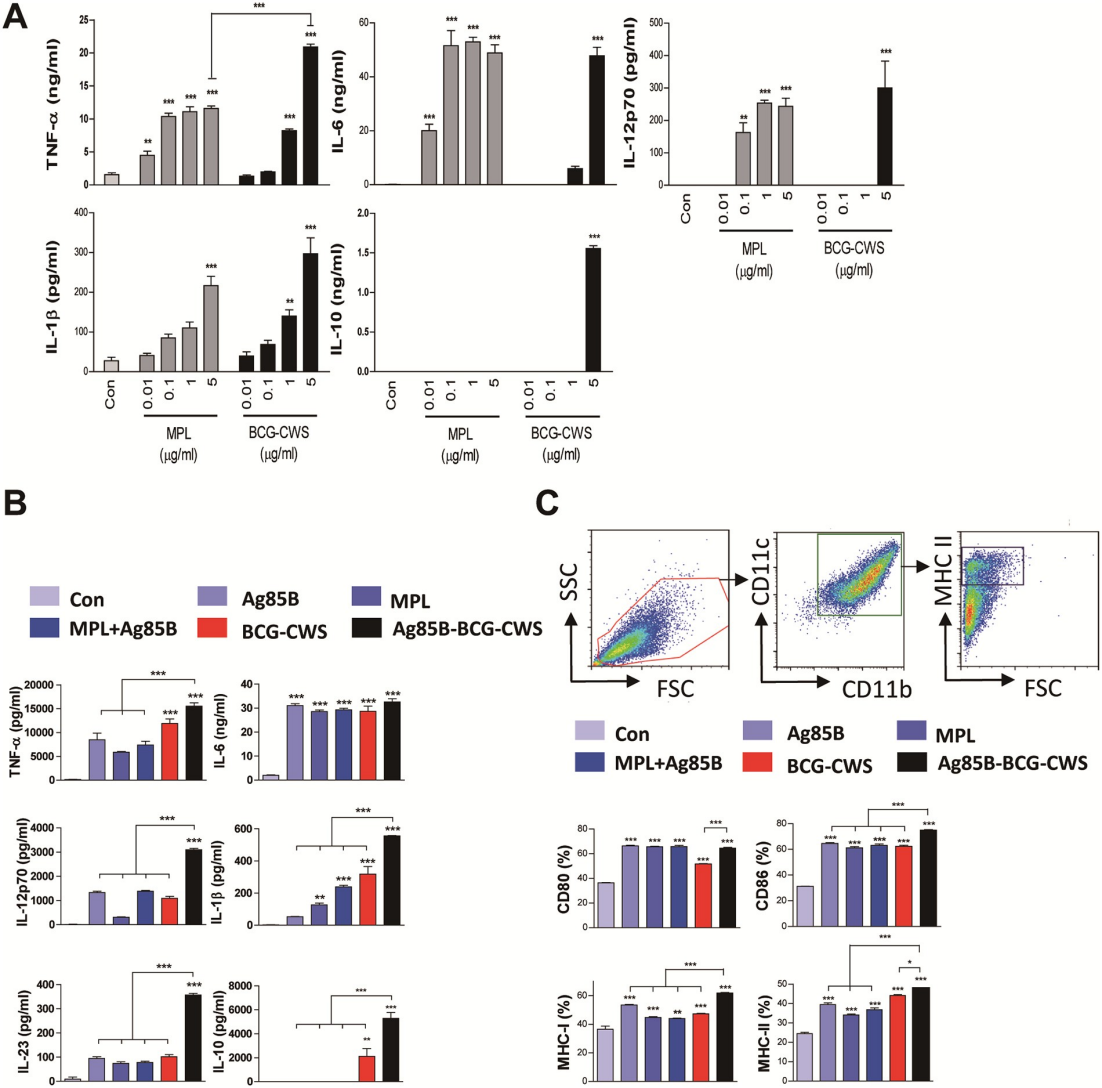
Ag85B-BCG-CWS induces DC maturation. (A) DCs were dose-dependently treated with the indicated concentrations of MPL or BCG-CWS for 24 h. TNF-α, IL-6, IL-1β, IL12p70, and IL-10 levels in the culture medium were measured by an ELISA. (B) DCs were treated with the indicated concentrations of Ag85B, MPL, MPL+Ag85B, BCG-CWS, or Ag85B-BCG-CWS for 24 h. TNF-α, IL-6, IL-1β, IL-12p70, IL-23, and IL-10 levels in the culture medium were measured by an ELISA. (C) DCs were stained with anti-CD80, anti-CD86, anti-MHC class I, or anti-MHC class II mAbs and analyzed for the expression of surface markers. The frequency of positive cells is shown for each panel. All bar graphs show the means ± SD of 3 samples. One representative plot out of three independent experiments is shown; **p < 0.01 or ***p < 0.001 compared to control group.

It has been demonstrated that the BCG-CWS induces the activation of human DCs [14, 21]. We first tested whether BCG-CWS could activate the mouse BMDCs and compared its activities with MPL, which is well known as a TLR-4 agonist. As shown in Fig 2A, DCs treated with MPL induced the higher production of the pro-inflammatory cytokines, than BCG-CWS at the low concentrations (0.01 to 1 μg/mL). However, BCG-CWS induced similar or significantly higher production of the cytokines in BMDCs compared to MPL at a concentration of 5 μg/mL. IL-1β production was similar between the DCs stimulated with MPL and BCG-CWS at all concentration tested. Unexpectedly, the BCG-CWS stimulated DCs to produce significant higher IL-10 production at the concentration of 5 μg/mL.

Next, we tested the activities of the BCG-CWS conjugated with Ag85B on BMDCs (Fig 2B). Production of IL-12p70, IL-1β, IL-10, and IL-23 were significantly higher in BMDCs stimulated with Ag85B-BCG-CWS than with BCG-CWS, Ag85B, MPL alone, or MPL+Ag85B (Fig 2B). The BCG-CWS-induced TNF-α production was not significantly enhanced by the Ag85B conjugation. There was no difference in IL-6 production among the stimulants.

We further analyzed the surface makers related to DC maturation by flow cytometry. Ag85B-BCG-CWS-treated DCs had significantly enhanced expression of CD86, MHC class I, and MHC class II molecules as compared to Ag85B, BCG-CWS, MPL, or MPL+Ag85B treated cells (Fig 2C). In particular, Ag85B or BCG-CWS activity was enhanced by Ag85B conjugation to BCG-CWS, but MPL-mediated activity was not enhanced by mixing with Ag85B.

### Ag85B-BCG-CWS-matured DCs induce Th1 and Th17 responses

**Fig 3:**
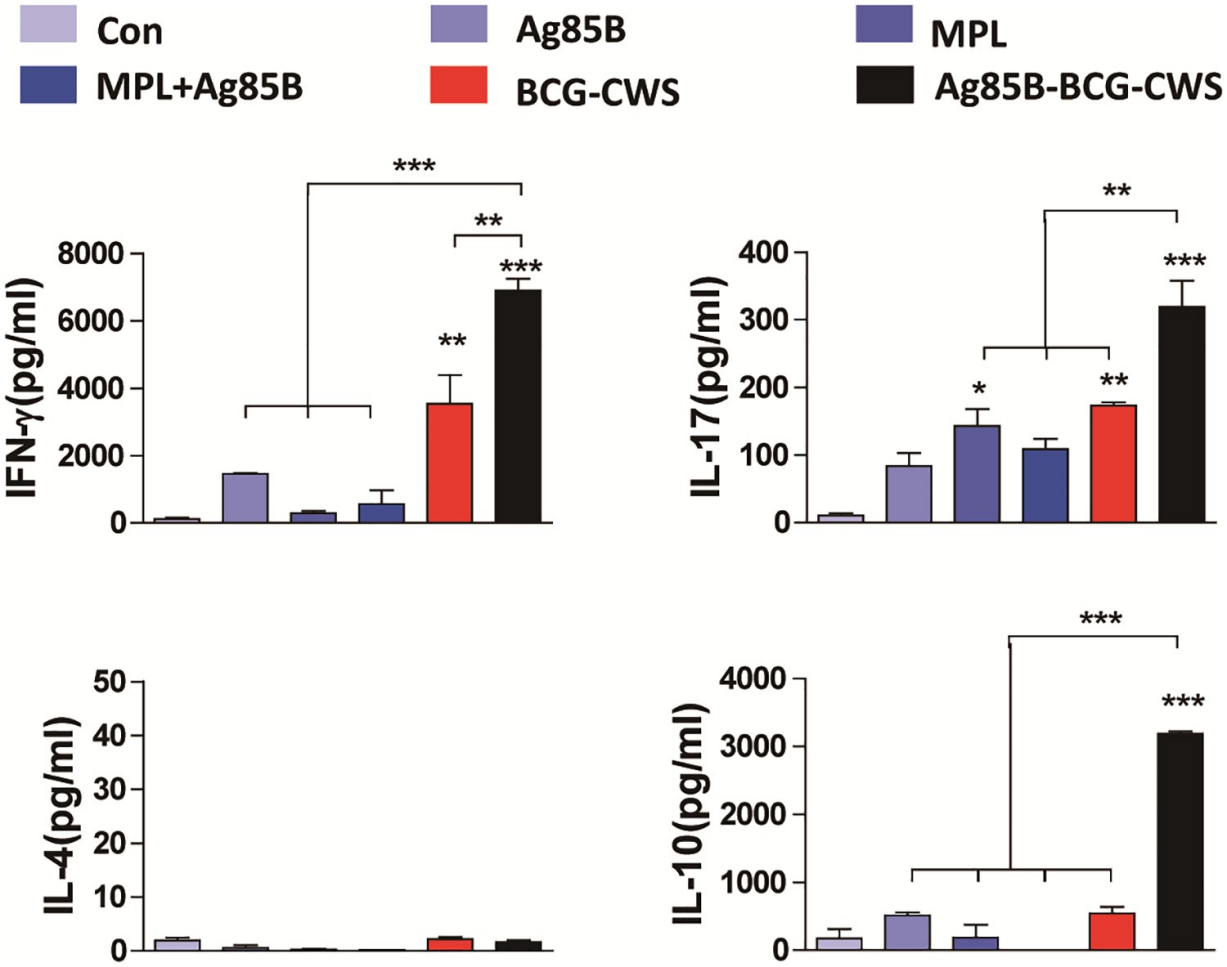
Ag85B-BCG-CWS-treated DCs stimulate T cells to produce Th1 and Th17 cytokines. T cells were purified from splenocytes of naive mice via MACS column. The T cells were co-cultured with unstimulated DCs, Ag85B (2.5 μg/ml)-stimulated DCs, MPL (5 μg/ml)-stimulated DC, MPL (5 μg/ml)+Ag85B (2.5 μg/ml)-stimulated, BCG-CWS (5 μg/ml)-stimulated DCs, or Ag85B (2.5 μg/ml)-BCG-CWS(5 μg/ml)-stimulated DCs for 5 days at a DC to T cell ratio of 1:10. The cytokine levels in the culture supernatants were measured by an ELISA. The data shown are the mean values ± SD (n = 3); *p < 0.05, **p < 0.01, and ***p < 0.001, compared to control group.

It is well known that IL-12p70, IL-1β, and IL-23 are involved in the induction of Th1 or Th17 responses [22]. Therefore, we investigated whether Ag85B-BCG-CWS-matured BMDCs could induce polarization to Th1 or Th17 responses. DCs matured with each antigen were co-cultured with syngeneic CD4^+^ T cells for 5 days and then the cytokines were quantified from the culture supernatants. BCG-CWS-treated DCs induced significantly higher IFN-γ production when compared to MPL-treated DCs (Fig 3). The production of IFN-γ and IL-17 was significantly enhanced in DCs treated with Ag85B-BCG-CWS as compared to DCs treated with MPL, MPL+Ag85B, BCG-CWS, or Ag85B alone. Ag85B-BCG-CWS induced IL-4 production, but their levels were not significant (Fig 3). Significantly higher IL-10 production was induced in Ag85B-BCG-CWS-treated DCs when compared to other antigen-stimulated DCs. Because IL-10 was produced from both the DCs and T cells, we performed FACS analysis to detect intracellular IL-10 in the co-cultured cells. As shown in S1 Fig, Ag85B-BCG-CWS-mediated IL-10 production occurred predominantly in DCs rather than T cells.

### Ag85B-BCG-CWS induces antigen-specific Th1 and Th17 responses in mice

First, we simply compared the protective efficacy of Ag85B+MPL-DDA and Ag85B-BCG-CWS in a short-term mouse challenge model. Mice were immunized, as indicated in S2A Fig, and then challenged with Mtb 4 weeks after the last immunization. Bacterial load was evaluated 6 weeks after infection. As shown in S2B Fig, the bacterial burden in the lungs and the spleen was significantly reduced in the Ag85B+MPL-DDA- and Ag85B-BCG-CWS-immunized groups, but not in the MPL-DDA- or BCG-CWS-immunized groups, when compared to the infection control. There was no significant difference in protective efficacy between Ag85B+MPL-DDA- and Ag85B-BCG-CWS-immunized mice, although the bacterial burden in Ag85B+MPL-DDA-immunized mice was slightly lower than in Ag85B-BCG-CWS-immunized mice. Therefore, in subsequent mouse experiments, we focused on Ag85B-BCG-CWS-mediated immune responses.

**Fig 4:**
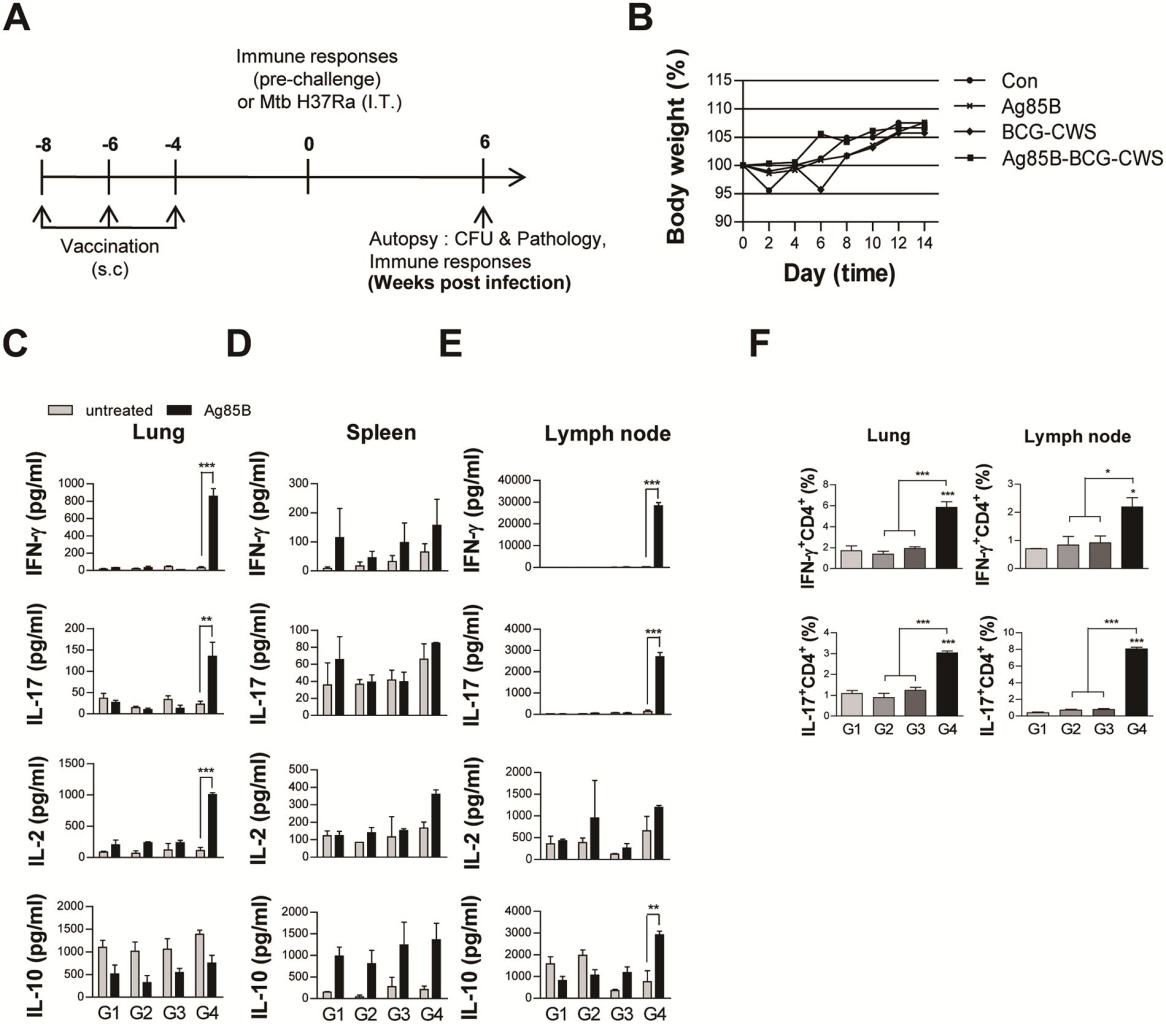
Ag-specific responses in lung cells and cervical lymph node 4 weeks after the final immunization with Ag85B alone, BCG-CWS alone, or Ag85B-BCG-CWS. (A) Schematic diagram of the experimental design. (B) Group of three female C57BL/6 mice were given a single subcutaneous injection with Ag85B alone, BCG-CWS alone, or Ag85B-BCG-CWS at day 0. Change in body weight and mortality were monitored for 2 weeks. (C-E) IFN-γ, IL-17, IL-2, and IL-10 production by lung cells, spleen, and cervical lymph node in response to untreated or Ag85B stimulation was measured by an ELISA. The data are shown as the means ± SD of 3 samples. One representative plot out of three independent experiments is shown. **p < 0.01 or ***p < 0.001 compared between the untreated and Ag85B-stimulated cells in each group. (F) Subsequently, IFN-γ-producing T Cells (IFN^+^CD4^+^) and IL-17-producing T cells (IL-17^+^CD4^+^) were counted by FACS. The gating strategy to assess intracellular cytokine are shown in S3A Fig. *p < 0.05 or ***p < 0.001 compared to group 1. All graphs show the results from one of the two experiments producing similar results (n = 3 animals/group). Group G1: naive control, G2: Ag85B alone, G3: BCG-CWS alone, and G4: Ag85B-BCG-CWS.

Next, we investigated whether the Ag85B-BCG-CWS could induce the Th1 or Th17 response *in vivo*. The C57BL/6 mice were immunized three times with Ag85B, BCG-CWS, or Ag85B-BCG-CWS (Fig 4A). Because one of the most important aspects of adjuvant development is safety, the mice were monitored for 2 weeks after the first immunization. There was no abnormal change or behavior in any of mice injected when compared to the non-injected control mice. Ag85B-BCG-CWS-injected mice showed no significant weight change during the 2-week observation, when compared to the mice injected with BCG-CWS or Ag85B alone and non-treated mice (Fig 4B). Next, we investigated the immune response against Ag85B in cells from lung, spleen, and lymph node at 4 weeks after the last immunization before the challenge. Production of IFN-γ, IL-17, IL-2, and IL-10 from the culture supernatants of the cells stimulated with Ag85B was determined. The production of IFN-γ and IL-17 in both the lung and lymph node, and IL-2 in the lungs were significantly increased in only the Ag85B-BCG-CWS-immunized mice (Fig 4C and 4E). The cytokine production in the spleens had no significant difference among the immunized groups (Fig 4D). Although the Ag85B-BCG-CWS-stimulated DCs produced significantly higher levels of IL-10 than other antigen-stimulated DCs (Fig 2), Ag85B-specific IL-10 production in the spleen and lung was not observed in all immunized groups, except in the lymph node of Ag85B-BCG-CWS-immunized mice (Fig 4C, 4D and 4E). FACS analysis also showed that the IFN-γ- or IL-17-positive CD4^+^ T cells significantly expanded in the lung and lymph node of the Ag85B-BCG-CWS-immunized mice, when compared to other immunized mice (Fig 4F). These data suggest that Ag85B-BCG-CWS can induce the Th1 and Th17 responses *in vivo* before challenge.

### Protective effect of Ag85B-BCG-CWS

**Fig 5:**
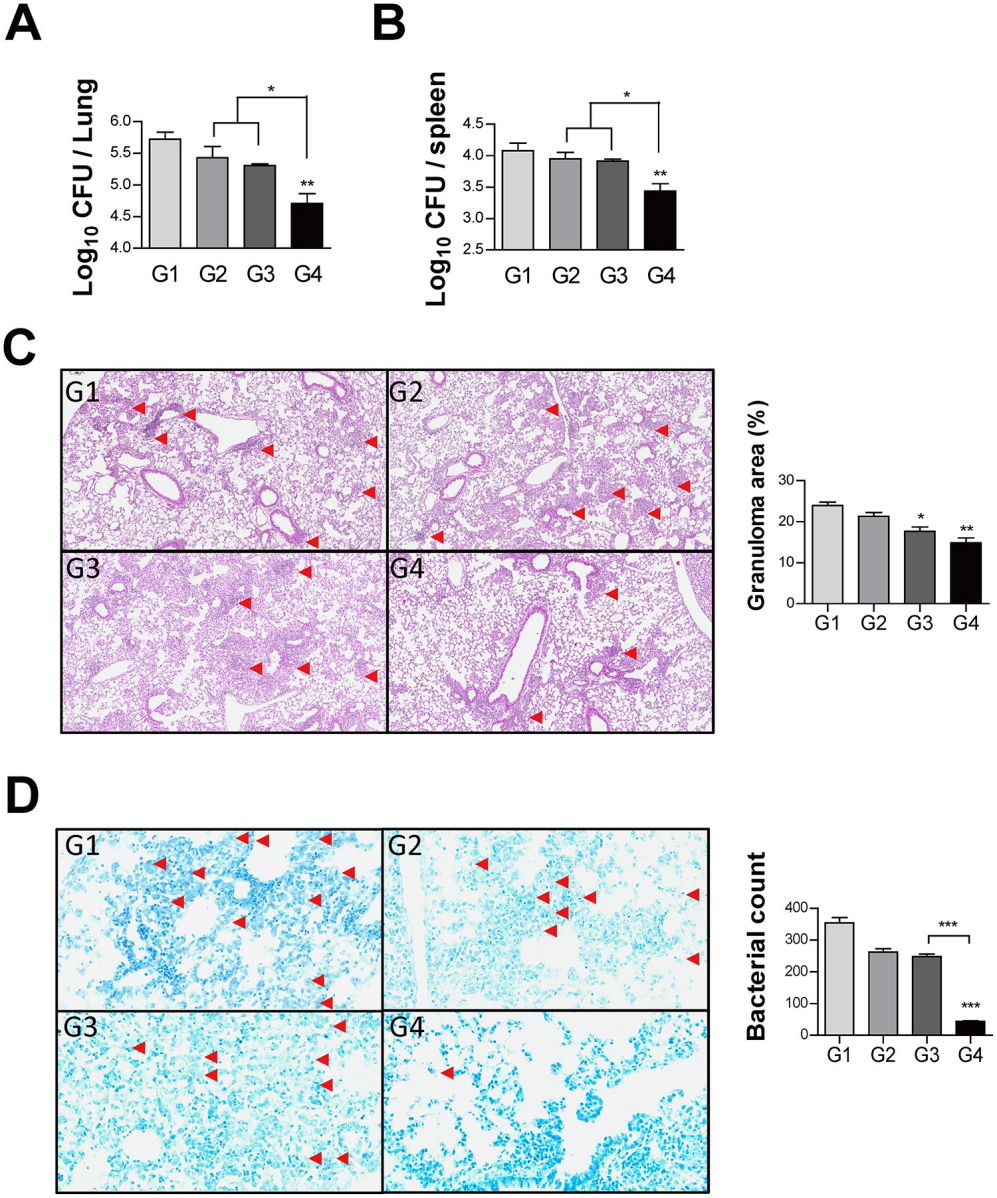
Histology of representative lung lobes and CFU values for each group. (A—B) Differences in the bacterial burden between mice immunized with Ag85B alone, BCG-CWS alone, or Ag85B-BCG-CWS at 6 weeks after challenge with Mtb are shown. Lung sections from each immunized mouse (immunization with Ag85B alone, BCG-CWS alone, or Ag85B-BCG-CWS) were stained with (C) H&E and (D) AFB at 6 weeks after the challenge with Mtb. The granuloma area (%) and the bacterial count from a lung section have been plotted. The results from one of the two experiments producing similar results are shown (n = 3 animals/group). *p < 0.05, **p < 0.01, and ***p < 0.001, compared to group 1. Group G1: Infection control, G2: Ag85B alone, G3: BCG-CWS alone, and G4: Ag85B-BCG-CWS. (H&E magnification 40X, AFB magnification 200X).

The mice were challenged with Mtb H37Ra 4 weeks after the last immunization, and the bacterial loads and histopathologic changes were evaluated 6 weeks after infection (Fig 4A). The bacterial burden in the lungs and the spleen was significantly reduced in the Ag85B-BCG-CWS-immunized group, but not in Ag85B or BCG-CWS-immunized group when compared to the infection control (Fig 5A and 5B), suggesting that Ag85B-BCG-CWS has significantly higher protective efficacy than Ag85B or BCG-CWS alone.

Hematoxylin and eosin (H&E) staining of the lung sections indicated improved lung inflammation in the Ag85B-BCG-CWS-immunized group compared to the infection control (Fig 5C). Ag85B-BCG-CWS showed significantly reduced bacterial count in tissue section stained for AFB, compared to the BCG-CWS or infection control (Fig 5D). In addition, BCG-CWS alone had a considerable protective efficacy on H&E and AFB stains of the lung tissue section.

**Fig 6:**
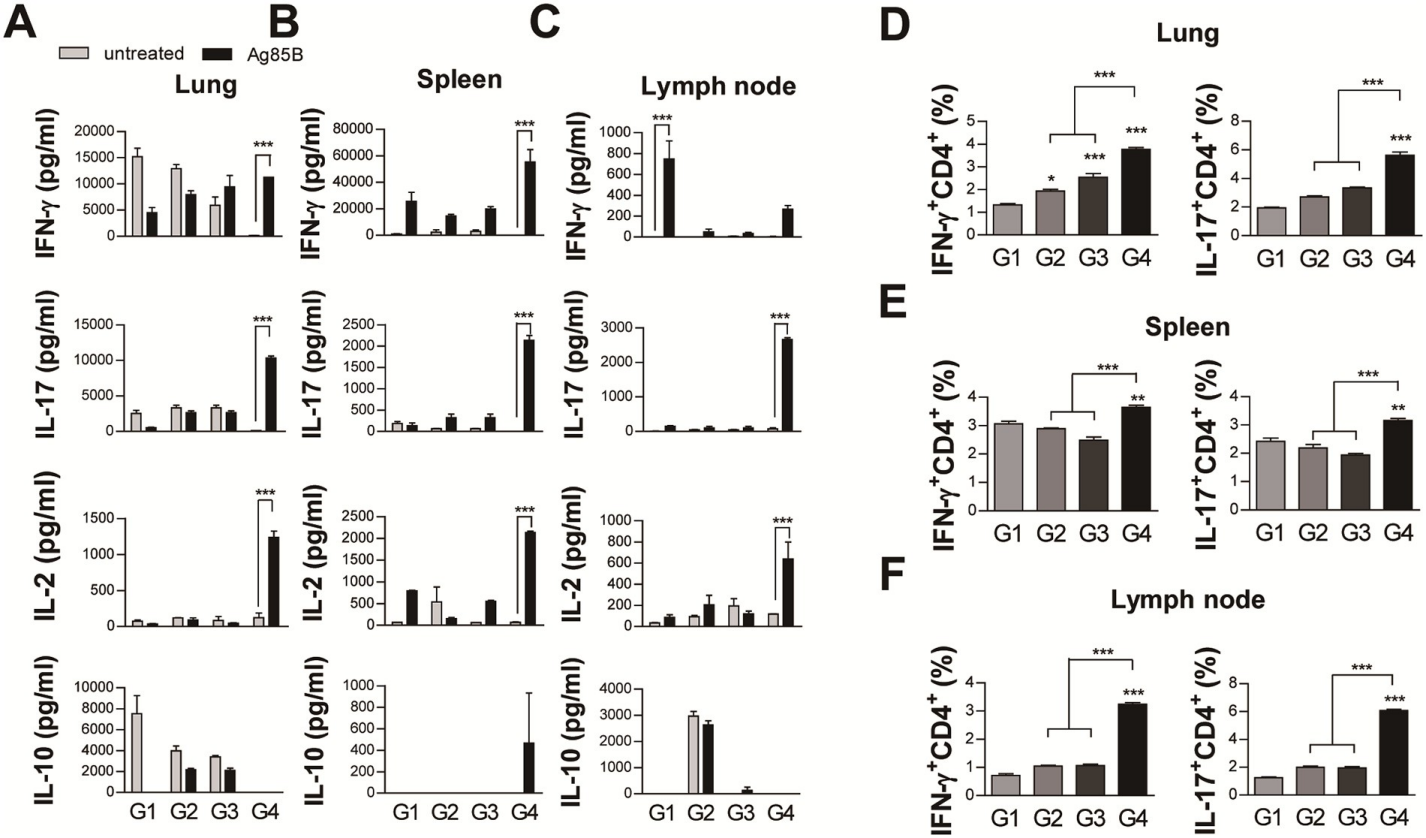
Ag85B-BCG-CWS induces Ag-specific Th1 and Th17 responses in lung cells and in the cervical lymph node after challenge with Mtb. (A-C) IFN-γ, IL-17, IL-2 and IL-10 production by lung cells, spleen, and cervical lymph node in response to untreated or Ag85B stimulation was measured by an ELISA. The data are shown as the means ± SD of 3 samples. One representative plot out of three independent experiments is shown. ***p < 0.001 compared to between the untreated and Ag85B-stimulated cells in each group. Subsequently, IFN-γ-producing T cells (IFN^+^CD4^+^) and IL-17-producing T cells (IL-17^+^CD4^+^) from (D) lung, (E) spleen, and (F) cervical lymph node were counted by FACS. The gating strategy to assess intracellular cytokine are shown in S3B Fig. *p < 0.05, **p < 0.01, and ***p < 0.001 compared to group 1. All graphs show the results from one of the two experiments producing similar results (n = 3 animals/group). Group G1: Infection control, G2: Ag85B alone, G3: BCG-CWS alone, and G4: Ag85B-BCG-CWS.

At 6 weeks post-challenge, we re-examined the Ag85B-specific immune response in the cells from the lungs, spleens, and cervical lymph nodes. Only the Ag85B-BCG-CWS-immunized group induced the Ag85B-specific IFN-γ and IL-17 immune responses in ELISA analysis (Fig 6A, 6B and 6C) and intracellular cytokine stain of CD4^+^ T cells (Fig 6B). Although a significant IFN-γ production was not observed only in the lymph nodes of Ag85B-BCG-CWS-immunized mice (Fig 6C), but IFN-γ^+^ CD4^+^ T cells were expanded in the lymph node as well as the lung and spleen (Fig 6D, 6E and 6F). Interestingly, the Ag85B-specific IL-2 production was observed in only the lungs before challenge (Fig 4C), but in all the tissues after challenge (6A, 6B, and 6C), in the Ag85B-BCG-CWS-immunized mice. There was no significant Ag85B-specific IL-10 production in any of the immunized groups after the infection.

### Long-term protective efficacy of Ag85B-BCG-CWS

**Fig 7:**
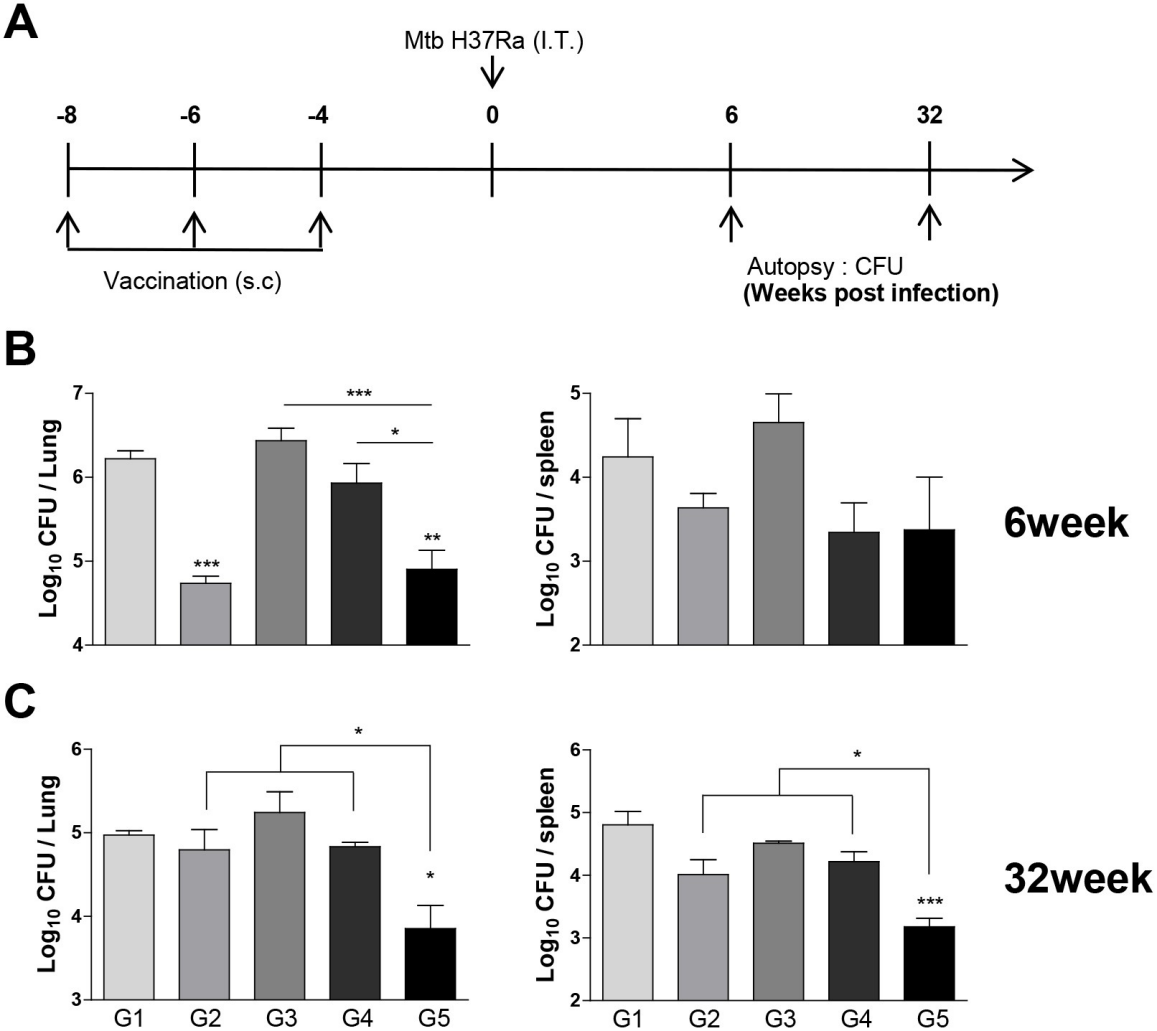
The long-term protective potential of Ag85B-BCG-CWS against Mtb challenge. (A) Schematic diagram of the experimental design. Eight weeks after the first vaccine injection, mice were challenged with virulent Mtb. (B) Six or (C) thirty-two weeks post-challenge, the mice were sacrificed and the bacterial burden (CFU) was measured in the lung and spleen. The results from one of the two experiments producing similar results are shown (n = 3 animals/group). *p < 0.05, **p < 0.01, and ***p < 0.001 compared to each to group 1. Group G1: Infection control, G2: BCG alone, G3: Heat-killed BCG, G4: BCG-CWS alone, and G5: Ag85B-BCG-CWS.

Finally, we determined the long-term protective efficacy of Ag85B-BCG-CWS and its efficacy was compared with live BCG or killed BCG. The mice were infected with Mtb H37Ra 4 weeks after the last immunization, and then CFUs in the lungs and the spleens were measured at 6 and 32 weeks after the infection (Fig 7A). When compared to the infection control or BCG-CWS immunization, the bacterial burden in the lungs 6 weeks post-infection was significantly reduced in the live BCG-injected group and in the Ag85B-BCG-CWS-immunized group; however, there was no significant difference between the two groups (Fig 7B). However, at 32 weeks after infection, only the Ag85B-BCG-CWS-immunized group showed a significant bacterial reduction in the lungs and spleens, and its protective efficacy was significantly higher than live BCG or BCG-CWS (Fig 7C). We tested the efficacy of the killed BCG because BCG-CWS was derived from the BCG cells. As expected, the killed BCG showed no protective effect.

## Discussion

It is a critical goal to develop an effective antigen delivery system in TB vaccine research field [7]. In general, adjuvants are mixed with the antigen as a form of emulsions or liposomes. However, these mixtures enable activation of surrounding APCs that do not present the delivered antigen and are probable cause of induction of autoimmune responses [23, 24]. In addition, a high adjuvant dosage is required to induce a satisfactory immune response to the antigen and leads to unnecessary side effects [25]. In contrast, an antigen-adjuvant conjugate can ensure that both the antigen and adjuvant reach the APC simultaneously, and strongly induce immune response, compared to an antigen-adjuvant mixture [26]. Recently, the antigen-adjuvant conjugation has attracted attention, because it can induce a stronger immune response at a lower concentration than the antigen-adjuvant mixture [26]. In this study, we demonstrated that the immunization of the BCG-CWS conjugated with Ag85B elicits Th1 and Th17 responses and a long-term protective response against TB in mice.

It is well known that mycobacterial components are typical immuno-adjuvants, such as the complete Freund’s adjuvant (CFA). BCG has been used in humans for the prevention of TB since 1921 and for immunotherapy against superficial bladder cancer [27], establishing their safety and immunogenicity. Therefore, BCG or its components are promising candidates to develop safe adjuvants or vehicle. We also previously demonstrated that BCG-CWS is an universal vaccine vehicle [11]. BCG-CWS consists of several components: mycolic acid, arabinogalactan, and a peptidoglycan complex [20, 28, 29]. Arabinogalactan is a biocompatible and mass-producible natural polysaccharide. Arabinogalactan increases splenocyte proliferation, stimulates macrophages, and enhances the relevant cytokine release [9, 30]. The arabinogalactan-poly(I:C) induces a strong Th1 response against the conjugated protein [9, 30]. Peptidoglycan activates DCs to secrete IL-23 and IL-1β via the TLR2 pathway and eventually differentiates naive T cells into Th17 memory cells [31]. The BCG-CWS promotes sufficient antibody production and exerts anti-tumor activity in immuno-adjuvant therapy, acting as a potent adjuvant [11]. BCG-CWS has been applied in the treatment of bladder cancer [12, 13]. BCG-CWS activates DCs to express the costimulatory molecules and to secret TNF-α, IL-6, and IL-12p40 through the TLR2 and TLR4 signaling pathways [14, 21]. Therefore, we hypothesized that, if BCG-CWS conjugated with a protein used as a TB vaccine, BCG-CWS can enhance the immune response specific to the conjugated protein. In this study, we found that BCG-CWS enhanced the Ag85B-mediated pro-inflammatory cytokine production and expression of co-stimulatory and MHC class I/II molecules, when DCs were stimulated with Ag85B-conjugated BCG-CWS.

It has been reported that the TB vaccine provides immunity by inducing antigen-specific Th1- and Th17-cell-based immunity. Our data indicated that Ag85B-BCG-CWS activated DCs to secrete TNF-α, IL-12, IL-1β, and IL-23, which are involved in either Th1 or Th17 differentiation. In fact, Ag85B-BCG-CWS-matured DCs induced both the Th1 and Th17 responses, which were also induced in the lungs, spleens, and lymph node from mice immunized with Ag85B-BCG-CWS before infection and these responses were maintained after challenge as well. Simultaneously, BCG-CWS or Ag85B-BCG-CWS significantly induced the anti-inflammatory cytokine IL-10 in DCs, but the Ag85B-specific IL-10 production in lungs from Ag85B-BCG-CWS-immunized mice was not observed. Early sources of IL-10 during infection are macrophages or DCs, but the major source of IL-10 production in adaptive cell-mediated response is the T cells [32]. Therefore, it appears that the T cells activated in the Ag85B-BCG-CWS-immunized mice might not produce IL-10 at high level.

IFN-γ plays a critical role in the control of Mtb infection, as indicated by the IFN-γ^-/-^ mice which are affected by disseminated TB infection [33]. Recently, it has been reported that IFN-γ is necessary to elicit the vaccine-specific CD4^+^ T cell response, but IFN-γ responsiveness is not essential for the control of the Mtb burden by vaccination [34]. It is also reported that there is an IFN-γ-independent mechanism for the control of Mtb infection *in vivo* [35], where they suggest the involvement of Th17 in TB protection. There has been conflicting reports on the protective roles of Th17/IL-17 responses during primary infection [36–38]. However, IL-17 contributes to the protection against TB in vaccine-induced immunity [39]. Recently, Gopal *et al*. reported that IL-17 provides mucosal vaccine-induced protection against TB via CXCL13 induction [40]. In this study, BCG-CWS enhanced the conjugated Ag85B-specific Th1 and Th17 responses in the lungs and lymph nodes before challenge and in the spleens too after challenge. In particular, significant expansion of IFN-γ^+^CD4^+^T cells or IL-17^+^ CD4^+^T cells was observed in Ag85B-BCG-CWS-immunized mice. These results suggest that BCG-CWS plays an essential role to induce both the Th1 and Th17 responses to the conjugated antigen.

In vaccine efficacy test, Ag85B-BCG-CWS showed significant protective effect in terms of bacterial loads and histopathology when compared to Ag85B or BCG-CWS alone. Although we previously demonstrated that H37Ra can be used to test vaccine efficacy in the mouse model [41], further investigation on the detailed protective efficacy and mechanism in a mouse model using virulent Mtb H37Rv is urgently needed. In comparing the test with BCG injection, Ag85B-BCG-CWS showed significant protective effect comparable with live BCG at 6 weeks after infection, but significant higher protective efficacy than live BCG, which had no protective effect, at 32 weeks after infection. Interestingly, BCG-CWS alone had some protective effect at 6 weeks after infection, but no protective effect at 32 weeks after infection. Because the CWS is a major component of the cell wall of BCG, it is possible that BCG-CWS alone could induce a short-term protective effect. H56 vaccine mixed with the adjuvant CFA01, which contains TDB as an immunomodulator, boosts the BCG with slightly lower bacterial counts than the BCG-vaccinated mice at 4 weeks after challenge; but 24 weeks after infection, H56 boosting results in significantly lower bacterial load than the BCG-vaccinated mice [42]. Taken together, our results suggest that BCG-CWS may be a reliable adjuvant for the subunit TB vaccine and is able to enhance a long-term protective response.

## Supporting information

S1 Fig Ag85B-BCG-CWS does not affect IL-10 secretion from T-cells. T cells activated by unstimulated DCs, Ag85B-stimulated DCs, MPL-stimulated DCs, MPL+Ag85B-stimulated DCs, BCG-CWS-stimulated DCs, or Ag85B-BCG-CWS-stimulated DCs were co-cultured for 1 day with CD4^+^ T cells of naïve mice at a DC-to-T-cell ratio of 1:10. (A) Subsequently, IL-10-producing DCs (CD11c^+^CD11b^+^IL-10^+^) (B) and IL-10-producing CD4^+^ T cells (CD4^+^IL-10^+^) were gated as shown.
(TIF)

S2 Fig Bacterial burden of Ag85B-BCG-CWS and Ag85B+MPL-DDA in mice. (A) Schematic diagram of the experimental design. (B) Differences in bacterial burden are shown among mice immunized with MPL (25 μg)-DDA (250 μg) alone, Ag85B (2.5 μg)+MPL (25 μg)-DDA (250 μg), BCG-CWS (5 μg) or Ag85B (2.5 μg)-BCG-CWS (5 μg) at 6 weeks after a challenge with Mtb H37Ra (n = 3 animals/group). *p < 0.05, **p < 0.01, and ***p < 0.001, compared with Infection only or Ag85B+MPL-DDA groups. Group 1 (G1): Infection only G2: MPL-DDA alone, G3: Ag85B+MPL-DDA, G4: BCG-CWS alone, G5: Ag85B-BCG-CWS. *dimethyl dioctadecyl ammonium bromide (DDA).
(TIF)

S3 Fig Gating strategy to assess intracellular cytokines. All samples were stained for surface molecules and gated based on forward scatter (FSC) and side scatter (SSC). T cells were gated from the lymphocyte gate by FSC vs. SSC, based on surface expression patterns of CD4^+^ T cells. Using the CD4^+^ T cell gate, cells with specific staining for IFN-γ and IL-17 are shown in stimulated spleen or lung cells. (A) lung cells and cervical lymph nodes, 4 weeks after final immunization (Fig 4F) and (B) 6 weeks after Mtb challenge (Fig 6D–6F).
(TIF)

## References

[1] Andersen P, Doherty TM. The success and failure of BCG—implications for a novel tuberculosis vaccine. Nature reviews Microbiology. 2005;3(8):656–62. 10.1038/nrmicro1211 . 16012514

[2] Wright A, Zignol M, Van Deun A, Falzon D, Gerdes SR, Feldman K, et al Epidemiology of antituberculosis drug resistance 2002–07: an updated analysis of the Global Project on Anti-Tuberculosis Drug Resistance Surveillance. Lancet. 2009;373(9678):1861–73. 10.1016/S0140-6736(09)60331-7 . 19375159

[3] Gandhi NR, Moll A, Sturm AW, Pawinski R, Govender T, Lalloo U, et al Extensively drug-resistant tuberculosis as a cause of death in patients co-infected with tuberculosis and HIV in a rural area of South Africa. Lancet. 2006;368(9547):1575–80. 10.1016/S0140-6736(06)69573-1 . 17084757

[4] Shah NS, Wright A, Bai GH, Barrera L, Boulahbal F, Martin-Casabona N, et al Worldwide emergence of extensively drug-resistant tuberculosis. Emerging infectious diseases. 2007;13(3):380–7. 10.3201/eid1303.061400 . 17552090 PMC2725916

[5] Calmette A. Preventive Vaccination Against Tuberculosis with BCG. Proceedings of the Royal Society of Medicine. 1931;24(11):1481–90. . 19988326 10.1177/003591573102401109 PMC2182232

[6] Reed SG, Orr MT, Fox CB. Key roles of adjuvants in modern vaccines. Nature medicine. 2013;19(12):1597–608. 10.1038/nm.3409 . 24309663

[7] Baldwin SL, Bertholet S, Reese VA, Ching LK, Reed SG, Coler RN. The importance of adjuvant formulation in the development of a tuberculosis vaccine. Journal of immunology. 2012;188(5):2189–97. 10.4049/jimmunol.1102696 . 22291184 PMC3288309

[8] van Dissel JT, Joosten SA, Hoff ST, Soonawala D, Prins C, Hokey DA, et al A novel liposomal adjuvant system, CAF01, promotes long-lived Mycobacterium tuberculosis-specific T-cell responses in human. Vaccine. 2014;32(52):7098–107. 10.1016/j.vaccine.2014.10.036 . 25454872

[9] Huang Q, Yu W, Hu T. Potent Antigen-Adjuvant Delivery System by Conjugation of Mycobacterium tuberculosis Ag85B-HspX Fusion Protein with Arabinogalactan-Poly(I:C) Conjugate. Bioconjugate chemistry. 2016;27(4):1165–74. 10.1021/acs.bioconjchem.6b00116 . 27002920

[10] Prados-Rosales R, Carreno L, Cheng T, Blanc C, Weinrick B, Malek A, et al Enhanced control of Mycobacterium tuberculosis extrapulmonary dissemination in mice by an arabinomannan-protein conjugate vaccine. PLoS pathogens. 2017;13(3):e1006250 10.1371/journal.ppat.1006250 . 28278283 PMC5360349

[11] Paik TH, Lee JS, Kim KH, Yang CS, Jo EK, Song CH. Mycobacterial cell-wall skeleton as a universal vaccine vehicle for antigen conjugation. Vaccine. 2010;28(50):7873–80. 10.1016/j.vaccine.2010.09.083 . 20937311

[12] Nakamura T, Fukiage M, Higuchi M, Nakaya A, Yano I, Miyazaki J, et al Nanoparticulation of BCG-CWS for application to bladder cancer therapy. Journal of controlled release: official journal of the Controlled Release Society. 2014;176:44–53. 10.1016/j.jconrel.2013.12.027 . 24389133

[13] Miyazaki J, Nishiyama H, Yano I, Nakaya A, Kohama H, Kawai K, et al The therapeutic effects of R8-liposome-BCG-CWS on BBN-induced rat urinary bladder carcinoma. Anticancer research. 2011;31(6):2065–71. . 21737624

[14] Tsuji S, Matsumoto M, Takeuchi O, Akira S, Azuma I, Hayashi A, et al Maturation of human dendritic cells by cell wall skeleton of Mycobacterium bovis bacillus Calmette-Guerin: involvement of toll-like receptors. Infection and immunity. 2000;68(12):6883–90. . 11083809 10.1128/iai.68.12.6883-6890.2000 PMC97794

[15] Kim WS, Kim JS, Cha SB, Kim H, Kwon KW, Kim SJ, et al Mycobacterium tuberculosis Rv3628 drives Th1-type T cell immunity via TLR2-mediated activation of dendritic cells and displays vaccine potential against the hyper-virulent Beijing K strain. Oncotarget. 2016;7(18):24962–82. 10.18632/oncotarget.8771 . 27097115 PMC5041883

[16] Han JM, Levings MK. Immune regulation in obesity-associated adipose inflammation. Journal of immunology. 2013;191(2):527–32. 10.4049/jimmunol.1301035 . 23825387

[17] Choi HG, Choi S, Back YW, Paik S, Park HS, Kim WS, et al Rv2299c, a novel dendritic cell-activating antigen of Mycobacterium tuberculosis, fused-ESAT-6 subunit vaccine confers improved and durable protection against the hypervirulent strain HN878 in mice. Oncotarget. 2017;8(12):19947–67. 10.18632/oncotarget.15256 . 28193909 PMC5386736

[18] Byun EH, Kim WS, Shin AR, Kim JS, Whang J, Won CJ, et al Rv0315, a novel immunostimulatory antigen of Mycobacterium tuberculosis, activates dendritic cells and drives Th1 immune responses. Journal of molecular medicine. 2012;90(3):285–98. 10.1007/s00109-011-0819-2 . 21993523

[19] Smith PF, Langworth TA, Mayberry WR, Houghland AE. Characterization of the membranes of Thermoplasma acidophilum. Journal of bacteriology. 1973;116(2):1019–28. . 4583228 10.1128/jb.116.2.1019-1028.1973 PMC285480

[20] Chatterjee D. The mycobacterial cell wall: structure, biosynthesis and sites of drug action. Current opinion in chemical biology. 1997;1(4):579–88. . 9667898 10.1016/s1367-5931(97)80055-5

[21] Uehori J, Matsumoto M, Tsuji S, Akazawa T, Takeuchi O, Akira S, et al Simultaneous blocking of human Toll-like receptors 2 and 4 suppresses myeloid dendritic cell activation induced by Mycobacterium bovis bacillus Calmette-Guerin peptidoglycan. Infection and immunity. 2003;71(8):4238–49. 10.1128/IAI.71.8.4238-4249.2003 . 12874299 PMC165983

[22] van Beelen AJ, Zelinkova Z, Taanman-Kueter EW, Muller FJ, Hommes DW, Zaat SA, et al Stimulation of the intracellular bacterial sensor NOD2 programs dendritic cells to promote interleukin-17 production in human memory T cells. Immunity. 2007;27(4):660–9. 10.1016/j.immuni.2007.08.013 . 17919942

[23] Annable T, Tomassian T, Jain S, Leibbrandt M, Cooke MP, Deane JA. Using Poly I:C as an adjuvant does not induce or exacerbate models of systemic lupus erythematosus. Autoimmunity. 2015;48(1):29–39. 10.3109/08916934.2014.959166 . 25483245

[24] Vera-Lastra O, Medina G, Cruz-Dominguez Mdel P, Jara LJ, Shoenfeld Y. Autoimmune/inflammatory syndrome induced by adjuvants (Shoenfeld’s syndrome): clinical and immunological spectrum. Expert review of clinical immunology. 2013;9(4):361–73. 10.1586/eci.13.2 . 23557271

[25] Amidi M, Romeijn SG, Verhoef JC, Junginger HE, Bungener L, Huckriede A, et al N-trimethyl chitosan (TMC) nanoparticles loaded with influenza subunit antigen for intranasal vaccination: biological properties and immunogenicity in a mouse model. Vaccine. 2007;25(1):144–53. 10.1016/j.vaccine.2006.06.086 . 16973248

[26] Slutter B, Soema PC, Ding Z, Verheul R, Hennink W, Jiskoot W. Conjugation of ovalbumin to trimethyl chitosan improves immunogenicity of the antigen. Journal of controlled release: official journal of the Controlled Release Society. 2010;143(2):207–14. 10.1016/j.jconrel.2010.01.007 . 20074597

[27] Martinez-Pineiro JA, Jimenez Leon J, Martinez-Pineiro L Jr, Fiter L, Mosteiro JA, Navarro J, et al Bacillus Calmette-Guerin versus doxorubicin versus thiotepa: a randomized prospective study in 202 patients with superficial bladder cancer. The Journal of urology. 1990;143(3):502–6. . 2106041 10.1016/s0022-5347(17)40002-4

[28] Brennan PJ, Nikaido H. The envelope of mycobacteria. Annual review of biochemistry. 1995;64:29–63. 10.1146/annurev.bi.64.070195.000333 . 7574484

[29] Azuma I, Ribi EE, Meyer TJ, Zbar B. Biologically active components from mycobacterial cell walls. I. Isolation and composition of cell wall skeleton and component P3. Journal of the National Cancer Institute. 1974;52(1):95–101. . 4590014 10.1093/jnci/52.1.95

[30] Nosalova G, Prisenznakova L, Paulovicova E, Capek P, Matulova M, Navarini L, et al Antitussive and immunomodulating activities of instant coffee arabinogalactan-protein. International journal of biological macromolecules. 2011;49(4):493–7. 10.1016/j.ijbiomac.2011.06.004 . 21689679

[31] Lin Y, Slight SR, Khader SA. Th17 cytokines and vaccine-induced immunity. Seminars in immunopathology. 2010;32(1):79–90. 10.1007/s00281-009-0191-2 . 20112107 PMC2855296

[32] Redford PS, Murray PJ, O’Garra A. The role of IL-10 in immune regulation during M. tuberculosis infection. Mucosal immunology. 2011;4(3):261–70. 10.1038/mi.2011.7 . 21451501

[33] Flynn JL, Chan J, Triebold KJ, Dalton DK, Stewart TA, Bloom BR. An essential role for interferon gamma in resistance to Mycobacterium tuberculosis infection. The Journal of experimental medicine. 1993;178(6):2249–54. . 7504064 10.1084/jem.178.6.2249 PMC2191274

[34] Orr MT, Windish HP, Beebe EA, Argilla D, Huang PW, Reese VA, et al Interferon gamma and Tumor Necrosis Factor Are Not Essential Parameters of CD4+ T-Cell Responses for Vaccine Control of Tuberculosis. The Journal of infectious diseases. 2015;212(3):495–504. 10.1093/infdis/jiv055 . 25637347 PMC4654754

[35] Gallegos AM, van Heijst JW, Samstein M, Su X, Pamer EG, Glickman MS. A gamma interferon independent mechanism of CD4 T cell mediated control of M. tuberculosis infection in vivo. PLoS pathogens. 2011;7(5):e1002052 10.1371/journal.ppat.1002052 . 21625591 PMC3098235

[36] Lyadova IV, Panteleev AV. Th1 and Th17 Cells in Tuberculosis: Protection, Pathology, and Biomarkers. Mediators of inflammation. 2015;2015:854507 10.1155/2015/854507 . 26640327 PMC4657112

[37] Khader SA, Pearl JE, Sakamoto K, Gilmartin L, Bell GK, Jelley-Gibbs DM, et al IL-23 compensates for the absence of IL-12p70 and is essential for the IL-17 response during tuberculosis but is dispensable for protection and antigen-specific IFN-gamma responses if IL-12p70 is available. Journal of immunology. 2005;175(2):788–95. . 16002675 10.4049/jimmunol.175.2.788

[38] Gopal R, Monin L, Slight S, Uche U, Blanchard E, Fallert Junecko BA, et al Unexpected role for IL-17 in protective immunity against hypervirulent Mycobacterium tuberculosis HN878 infection. PLoS pathogens. 2014;10(5):e1004099 10.1371/journal.ppat.1004099 . 24831696 PMC4022785

[39] Khader SA, Bell GK, Pearl JE, Fountain JJ, Rangel-Moreno J, Cilley GE, et al IL-23 and IL-17 in the establishment of protective pulmonary CD4+ T cell responses after vaccination and during Mycobacterium tuberculosis challenge. Nature immunology. 2007;8(4):369–77. 10.1038/ni1449 . 17351619

[40] Gopal R, Rangel-Moreno J, Slight S, Lin Y, Nawar HF, Fallert Junecko BA, et al Interleukin-17-dependent CXCL13 mediates mucosal vaccine-induced immunity against tuberculosis. Mucosal immunology. 2013;6(5):972–84. 10.1038/mi.2012.135 . 23299616 PMC3732523

[41] Choi HG, Choi S, Back YW, Park HS, Bae HS, Choi CH, et al Mycobacterium tuberculosis Rv2882c Protein Induces Activation of Macrophages through TLR4 and Exhibits Vaccine Potential. PloS one. 2016;11(10):e0164458 10.1371/journal.pone.0164458 . 27711141 PMC5053528

[42] Aagaard C, Hoang T, Dietrich J, Cardona PJ, Izzo A, Dolganov G, et al A multistage tuberculosis vaccine that confers efficient protection before and after exposure. Nature medicine. 2011;17(2):189–94. 10.1038/nm.2285 . 21258338

