# Supplementary figures and images for: Cell wall skeleton of Mycobacterium bovis BCG enhances the vaccine potential of antigen 85B against tuberculosis by inducing Th1 and Th17 responses
Source: PLoS One. 2019 Mar 8;14(3):e0213536. doi: 10.1371/journal.pone.0213536 (PMC6407753; doi:10.1371/journal.pone.0213536)

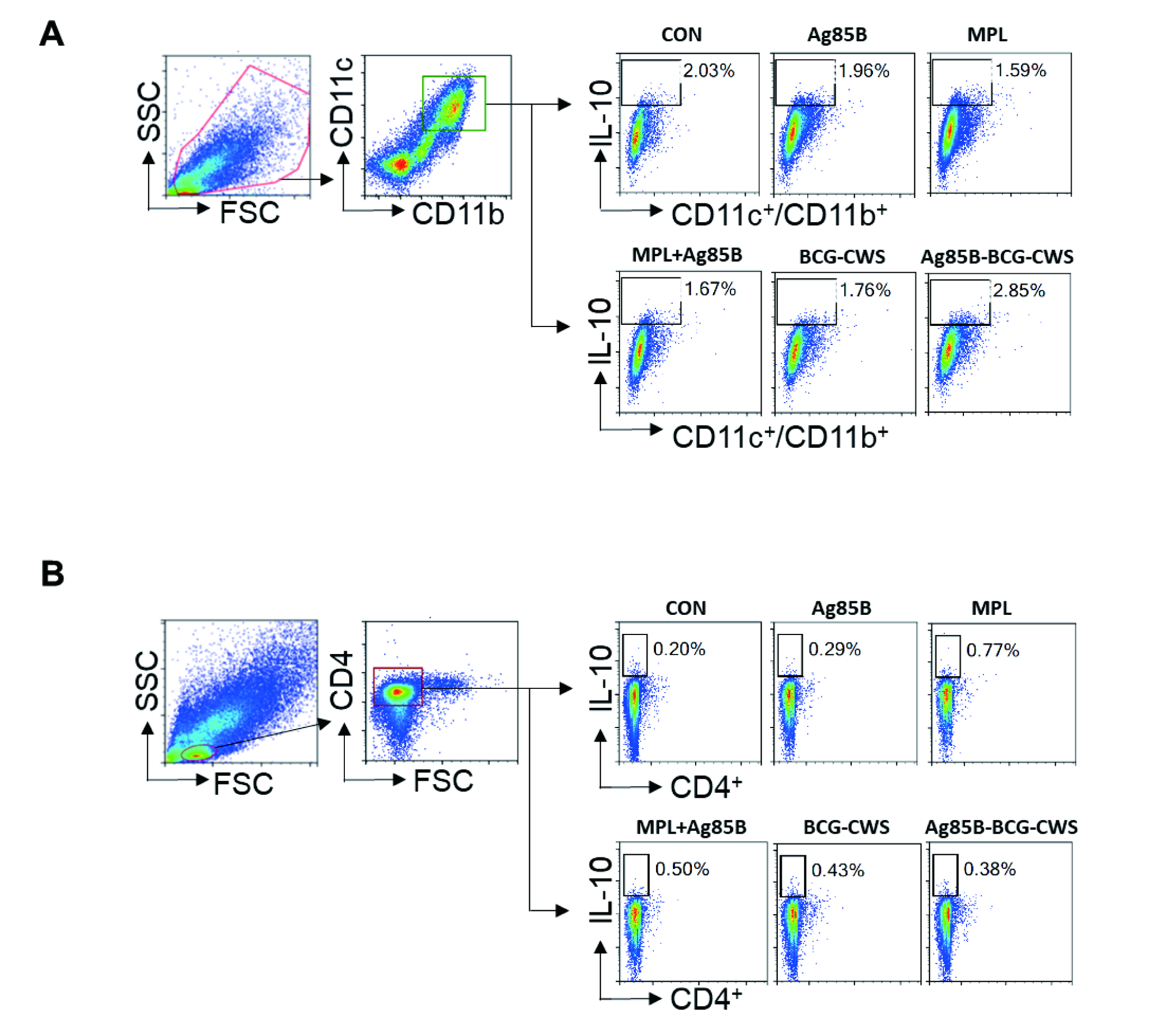

Supplement: S1 Fig — T cells activated by unstimulated DCs, Ag85B-stimulated DCs, MPL-stimulated DCs, MPL+Ag85B-stimulated DCs, BCG-CWS-stimulated DCs, or Ag85B-BCG-CWS-stimulated DCs were co-cultured for 1 day with CD4+ T cells of naïve mice at a DC-to-T-cell ratio of 1:10. (A) Subsequently, IL-10-producing DCs (CD11c+CD11b+IL-10+) (B) and IL-10-producing CD4+ T cells (CD4+IL-10+) were gated as shown. (TIF) [file pone.0213536.s001.tif]

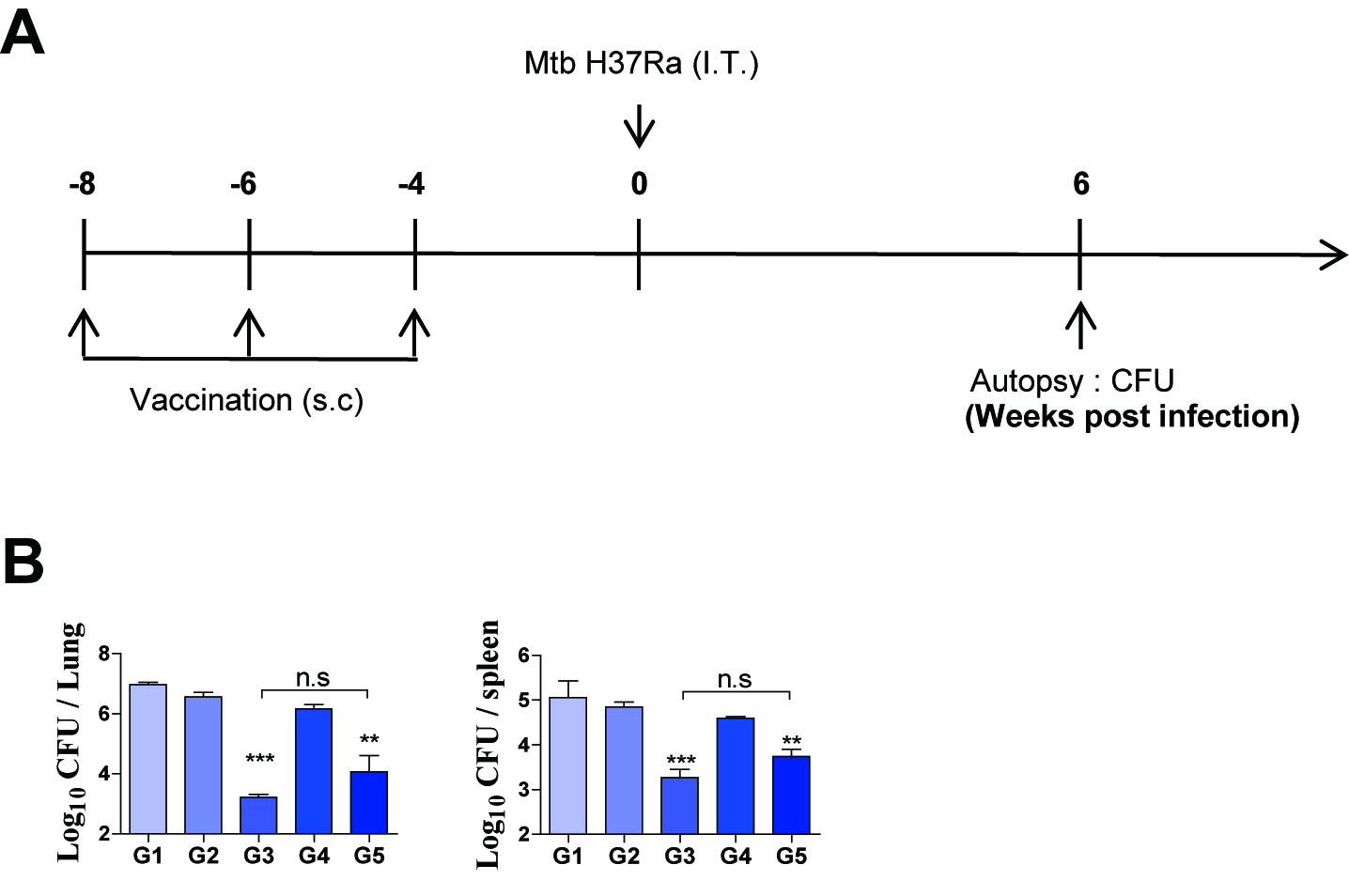

Supplement: S2 Fig — (A) Schematic diagram of the experimental design. (B) Differences in bacterial burden are shown among mice immunized with MPL (25 μg)-DDA (250 μg) alone, Ag85B (2.5 μg)+MPL (25 μg)-DDA (250 μg), BCG-CWS (5 μg) or Ag85B (2.5 μg)-BCG-CWS (5 μg) at 6 weeks after a challenge with Mtb H37Ra (n = 3 animals/group). *p < 0.05, **p < 0.01, and ***p < 0.001, compared with Infection only or Ag85B+MPL-DDA groups. Group 1 (G1): Infection only G2: MPL-DDA alone, G3: Ag85B+MPL-DDA, G4: BCG-CWS alone, G5: Ag85B-BCG-CWS. *dimethyl dioctadecyl ammonium bromide (DDA). (TIF) [file pone.0213536.s002.tif]

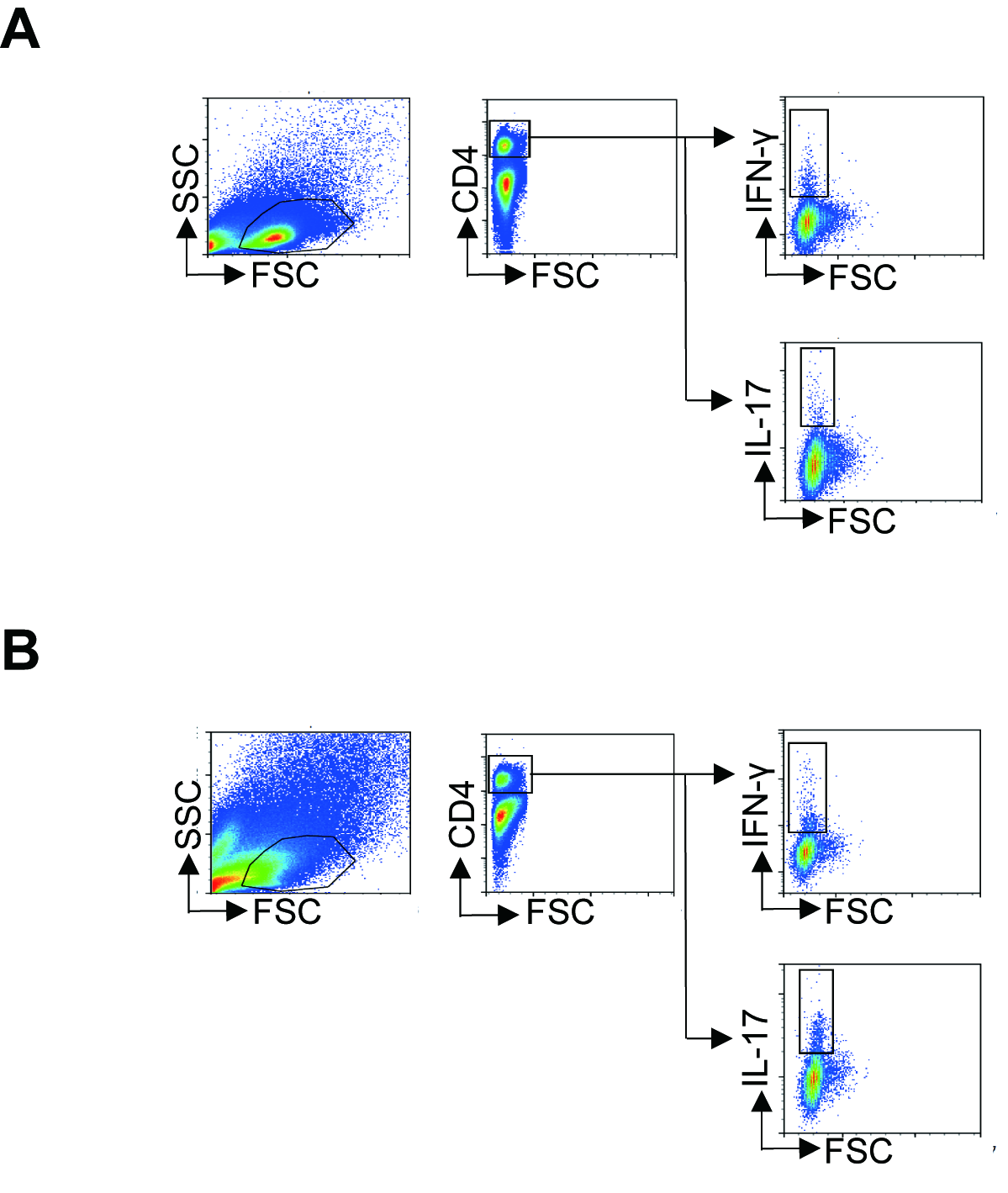

Supplement: S3 Fig — All samples were stained for surface molecules and gated based on forward scatter (FSC) and side scatter (SSC). T cells were gated from the lymphocyte gate by FSC vs. SSC, based on surface expression patterns of CD4+ T cells. Using the CD4+ T cell gate, cells with specific staining for IFN-γ and IL-17 are shown in stimulated spleen or lung cells. (A) lung cells and cervical lymph nodes, 4 weeks after final immunization (Fig 4F) and (B) 6 weeks after Mtb challenge (Fig 6D–6F). (TIF) [file pone.0213536.s003.tif]
